# Differences in the factors associated with tongue pressure between children with class I and Class II malocclusions

**DOI:** 10.1186/s12887-021-02956-x

**Published:** 2021-10-28

**Authors:** Yuko Fujita, Yoma Ohno, Keitaro Ohno, Tomohiro Takeshima, Kenshi Maki

**Affiliations:** Division of Developmental Stomatognathic Function Science, Department of Health Promotion, Kyushu Dental University, 2-6-1 Manazuru, Kokurakita-ku, 803-8580 Kitakyushu, Japan

**Keywords:** Malocclusion, Children, Tongue pressure, Masticatory performance

## Abstract

**Background:**

The relationship between tongue pressure and masticatory performance during the mixed dentition period in cases of Class II malocclusion has not been clarified. The aim of this study was to determine differences in tongue pressure-related factors, including maxillofacial morphology and masticatory performance, between Class I and Class II malocclusions during the mixed dentition period.

**Methods:**

A total of 56 children with Class I malocclusion (12 boys, 16 girls) or Class II malocclusion (16 boys, 12 girls) with mixed dentition were included in the present study. Height, body weight, hand grip strength, maximum occlusal force, maximum tongue pressure, masticatory performance, and the number of decayed, missing, and filled teeth were measured in all participants. Their lateral cephalograms were also evaluated. The means of all measurements were compared between Class I and Class II malocclusions. Pearson’s correlation coefficients were used to determine associations between maximum tongue pressure and other variables for each type of malocclusion.

**Results:**

The maximum tongue pressure, hand grip strength, and maximum occlusal force in the Class II malocclusion group were significantly lower than those in the Class I malocclusion group (all, *p* < 0.05). The maximum tongue pressure was significantly positively correlated with hand grip strength, maximum occlusal force, masticatory performance, and SNB (sella, nasion, B point) angle in the Class I group (all, *p* < 0.05), and with height, body weight, and labial inclination of the central incisors in the Class II group (all, *p* < 0.05).

**Conclusions:**

The maxillofacial morphometric factors associated with tongue pressure were clearly different between cases of Class I and Class II malocclusion with mixed dentition. Masticatory performance and tongue pressure were significantly positively correlated in cases of Class I malocclusion, but not in cases of Class II malocclusion.

## Background

Over the last few decades, problems related to the developmental deficiency of oral function in children all over the world have attracted a great deal of attention. A number of instruments have been developed for the objective evaluation of oral function, and many clinical studies of oral function have been reported [1–4].

Recent clinical studies reported that oral function undergoes significant development during the mixed dentition period [5, 6]. Several studies showed that the maximum tongue pressure peaks during the mixed dentition period, especially in girls [6], and tongue pressure in women decreases slowly with age from the 20 s [7]. These results suggest that the maximal acquisition of tongue pressure during the mixed dentition period may contribute to the suppression of future tongue pressure reduction.

Generally, changes in tongue pressure have been suggested to be associated with changes in dentition position and occlusion [8]. A previous study reported that patients with an anterior open bite and tongue thrusting at swallowing exhibited weaker maximum tongue pressure than did healthy adults [9]. A recent study showed that the maximum tongue pressure in cases of Class II malocclusion was significantly lower than in cases of Class I and Class III malocclusions in children around 9 years old [10].

Therefore, we hypothesized that each type of malocclusion has its own factors associated with tongue pressure, as each type of malocclusion has different dental and maxillofacial morphological characteristics. However, little is known about the factors associated with tongue pressure specific to Class II malocclusion during the mixed dentition period.

Previous studies reported that malocclusion reduced masticatory performance in children and adults [11, 12]. One study showed that the masticatory performance was clearly reduced in cases of Class III malocclusion, followed in order by Class II and Class I malocclusions [12]. Another study reported that masticatory performance did not differ significantly between children with normal occlusion and those with Class II malocclusion [13].

On the other hand, several studies reported that the maximum tongue pressure in children is closely related to masticatory performance [6, 14]. However, the relationship between tongue pressure and masticatory performance in cases of Class II malocclusion during the mixed dentition period has not been clarified.

This study was performed to identify differences in tongue pressure-related factors, including maxillofacial morphology and masticatory performance, between cases of Class I and Class II malocclusion during the mixed dentition period.

## Materials and methods

### Participants

 This study was approved by the Human Investigation Committee of Kyushu Dental University (approval number: 18–37), and all participants and their parents/guardians provided written informed consent for participation in the study. The participants were recruited following an initial examination at a single private dental clinic in Japan.

A previous study reported that the mean maximum tongue pressure in children around 9 years of age was 36 ± 5.0 kPa [10]. If the true difference in mean maximum tongue pressure between cases of Class I and Class II malocclusion were 4 kPa, it would be necessary to include 26 participants in each group to be able to reject the null hypothesis that the means of the Class I and Class II groups are equal with 80 % power. The probability of Type I error associated with the test of this null hypothesis was 0.05.

The inclusion criteria were children at Hellman developmental stage III A (complete eruption of permanent first molar and incisors) with bilateral Class I or II molar and deciduous canines relationship in centric occlusion. The exclusion criteria were systemic disturbances, treatment with medications that could interfere directly or indirectly with muscular activity, and uncooperative behavior. In addition, children were excluded if they had disorders in oral tissue morphology or the structure or number of teeth, a negative overjet, a negative overbite, or a history of orthodontic treatment or temporomandibular dysfunction. Based on these criteria, the study population consisted of 56 children aged 7–10 years (Class I group, 12 boys and 16 girls; Class II group, 16 boys and 12 girls).

### Anthropometry and dental examination

Height and body weight were measured in the consultation room of the clinic. Height was measured to an accuracy of ± 0.1 cm using a portable digital stadiometer (AD-653; A&D, Tokyo, Japan), with the head in the Frankfort plane, whereas body weight was measured with an accuracy of 0.1 kg [14].

During the intraoral examination, the total number of decayed, missing, and filled teeth (DMFT index) for each patient was recorded [15].

### Hand grip strength

A portable grip strength meter (T-2288; Toei Light Co. Ltd., Saitama, Japan) was used to measure hand grip strength. Participants were asked to stand and hold a dynamometer in their hand with the arm parallel to the body, without squeezing the arm against the body. Hand grip strength was measured, in kg, twice for each hand (alternately) with a 30-s interval between trials. The highest value from either the left or right hand was recorded as the grip strength [16].

## Maximum occlusal force

Maximum occlusal force was measured using a portable occlusal force meter (GM10; Nagano Keiki Co., Ltd., Tokyo, Japan). Participants were instructed to bite the element with maximal voluntary muscular effort using their first molars. Maximum occlusal force was measured on each side, with a 30-s interval between bite measurements. The larger value from either the left or right side was recorded as the maximum bite force [16].

### Maximum tongue pressure

A tongue pressure manometer (JMS, Hiroshima, Japan) was used to measure maximum tongue pressure. Participants were examined while relaxed in a sitting position and were asked to place a balloon on the anterior part of their palate and close their lips, biting a hard ring with the upper and lower incisors. Then, they were asked to raise their tongues and press the balloon against the palate with maximal voluntary muscular effort for approximately 7 s. The pressure was measured in kPa using a digital voltmeter attached to the tongue pressure manometer [14].

### Masticatory performance

Masticatory performance was determined by measuring the concentration of dissolved glucose obtained from a cylindrical-shaped gummy jelly (GLUCOLUMN; GC Co. Ltd., Tokyo, Japan) with a glucose-measuring device (GLUCO SENSOR GS-II; GC Co. Ltd.). Prior to the test, participants were instructed on how to perform chewing movements and mouth rinsing procedures to prevent swallowing. The participants were then instructed to chew the gummy jelly for 20 s on the habitual chewing side. Previously, the habitual masticatory side was found to exhibit better masticatory performance than the non-habitual masticatory side [17]. Since masticatory efficacy can be influenced by various factors related to occlusion, we used a method in which masticatory efficiency was maximized. After chewing, the participants were asked to take 10 ml of distilled water into their mouth and to spit out the gummy jelly and distilled water into a filter cup. Then, the glucose concentration in the filtrate (mg/dl) was measured using a dedicated device [16].

### Cephalometry

**Fig. 1:**
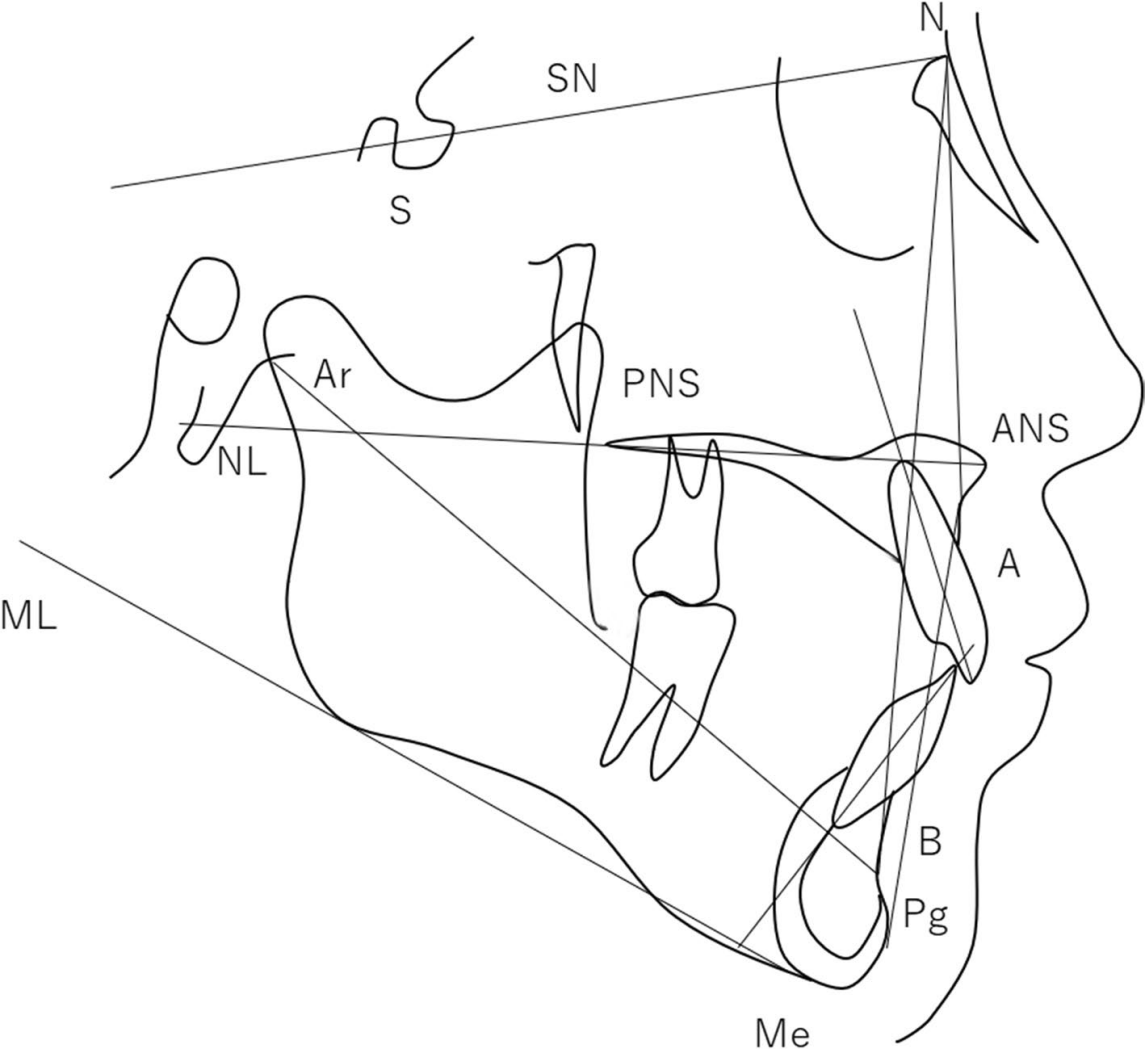
Reference lines and points in lateral cephalometric analysis. Measurement items; SNA angle (°), SNB angle (°), ANB angle (°), SN/ML angle (°), ML/NL angle (°), SN/NL angle (°), U1/NL angle (°), L1 to APg distance (mm), L1/ML (°), Interincisal angle (°), Ar to B distance (mm), Overjet (mm), and Overbite (mm).

Lateral cephalometric radiographs were taken using a Hyper-G/CM NEO PREMIUM system (ASAHIROENTGEN Ind. Co., Ltd., Kyoto, Japan) in accordance with the manufacturer’s instructions and traced. Cephalometric reference points and measurements were assessed according to the method of Schutz-Fransson et al. [18]. Reference lines and points are shown in Fig. 1.

### Reliability of measurements

With the exception of the lateral cephalometric radiographs, all examinations were repeated after a break of at least 30 min, and the mean values were used for subsequent analyses. The lateral cephalograms were performed twice at an interval of 2 weeks, and the mean values were used in the statistical analysis. All examinations were performed by the same examiner. Data generated during sample collection were assessed for reliability. Random error was characterized based on intraobserver reliability, which was quantified using the intraclass correlation coefficient (ICC). The data were assessed in terms of intraobserver reliability based on the ICC, where 0.800 ≤ ICC ≤ 1.000 corresponded to high reliability [19].

### Statistical analysis

All data were analyzed using SPSS for Windows (version 23.0; IBM Japan, Tokyo, Japan). The Shapiro–Wilk test was used to determine the normality of the data. All data for each group are expressed as the means ± standard deviations. Statistical comparisons between the groups were performed using two-way analysis of variance or the two-tailed *t* test. We used two-way analysis of variance to compare the measurements by sex (boys vs. girls) and occlusal relationship (Class I vs. Class II). Pearson’s correlation coefficients were used to determine associations between maximum tongue pressure and other variables. In the Class II group, partial correlations were used to assess the relationship between maximum tongue pressure and hand grip strength, while controlling for height and body weight. In all analyses, *p* < 0.05 was taken to indicate statistical significance.

## Results

The ICCs for all measurement items ranged from 0.81 to 0.92, indicating a high degree of intraobserver reliability.

Data on anthropometric measurements, hand grip strength, the DMFT index, maximum occlusal force, maximum tongue pressure, and masticatory performance are summarized in Table 1. Hand grip strength, maximum occlusal force, and maximum tongue pressure in the Class II group were significantly lower than those in the Class I group (*p* = 0.014, *p* = 0.011, and *p* = 0.000, respectively). There were no significant differences between the sexes in any parameter.

**Table 1:** Comparison of the sex and classification of malocclusion on measurements

	Class I			Class II				Two-Way ANOVA		
	**Boys** **(N = 12)**	**Girls** **(N = 16)**	**Total** **(N = 28)**	**Boys** **(N = 16)**		**Girls** **(N = 12)**	**Total** **(N = 28)**	**(Effects)** ***P*** **value**		
	**Mean (SD)**	**Mean (SD)**	**Mean (SD)**	**Mean (SD)**		**Mean (SD)**	**Mean (SD)**	**Boys** vs. **Girls**		**Class I** vs. **Class II**
Age (years)	8.88 (0.98)	9.06 (0.57)	8.98 (0.76)	8.73 (0.86)		8.97 (0.92)	8.83 (0.87)	0.366		0.594
Height (cm)	132.28 (7.80)	134.84 (8.80)	133.74 (8.33)	130.83 (10.21)		130.37 (6.44)	130.63 (8.65)	0.652		0.208
Body weight (kg)	29.49 (4.45)	31.34 (5.61)	30.55 (5.14)	30.05 (6.84)		27.74 (3.55)	29.06 (5.70)	0.875		0.305
Hand grip strength (kg)	14.67 (2.42)	14.06 (3.86)	14.32 (3.28)	12.25 (3.75)		11.83 (2.95)	12.07 (3.38)	0.578		0.014
DMFT index	5.17 (4.13)	2.06 (3.11)	3.39 (3.84)	3.94 (3.45)		4.00 (2.66)	3.96 (3.08)	0.100		0.698
Maximum occlusal force (kN)	0.44 (0.13)	0.40 (0.16)	0.42 (0.15)	0.34 (0.12)		0.30 (0.14)	0.33 (0.13)	0.335		0.011
Maximum tongue pressure (kPa)	38.31 (6.22)	36.81 (7.28)	37.45 (6.77)	32.33 (7.86)		27.79 (7.19)	30.39 (7.78)	0.128		0.000
Masticatory performance (mg/dl)	148.08 (38.23)	101.94 (46.31)	121.72 (48.24)	101.19 (44.99)		102.58 (38.50)	101.78 (41.58)	0.058		0.050

DMFT, decayed, missing, and filled teeth; SD, standard deviation; ANOVA, analysis of variance

According to the lateral cephalometric analysis, the ANB (A point, nasion, B point) angle and overbite were significantly higher in the Class II group than in the Class I group (*p* = 0.00 and *p* = 0.037, respectively; Table 2). The U1/NL angle was significantly smaller in the Class II group than in the Class I group (*p* = 0.022; Table 2).

**Table 2:** Comparison of the classification of malocclusions on the lateral cephalometric measurements

	Class I	Class II		
	**N = 28**	** N = 28**		
	**Mean (SD)**	**Mean (SD)**	***P*** **value**	
SNA angle (°)	79.16 (3.17)	80.25 (3.16)	0.203	
SNB angle (°)	76.88 (2.95)	75.55 (2.54)	0.078	
ANB angle (°)	2.28 (1.91)	4.70 (2.01)	0.000	
SN/ML angle (°)	36.49 (5.74)	36.97 (4.64)	0.731	
ML/NL angle (°)	28.61 (4.44)	28.68 (4.64)	0.953	
SN/NL angle (°)	7.88 (2.88)	8.29 (2.83)	0.592	
U1/NL angle (°)	116.77 (5.46)	112.27 (8.47)	0.022	
L1/ML angle (°)	95.14 (5.65)	95.25 (7.28)	0.951	
Interincisal angle (°)	120.73 (8.29)	125.79 (12.50)	0.080	
L1 to APg distance (mm)	3.89 (2.25)	2.89 (2.17)	0.096	
Ar to B distance (mm)	95.75 (6.45)	92.89 (5.40)	0.078	
Overjet (mm)	3.39 (1.78)	4.03 (1.48)	0.145	
Overbite (mm)	2.54 (1.23)	3.38 (1.69)	0.037	

SD, standard deviation

The results of Pearson’s correlation analysis for each variable are shown in Table 3.

**Table 3:** Pearson’s correlation coefficients for measurements among Class I and Class II participants

	Maximum tongue pressure		
	**Class I**	**Class II**	
Height	0.333	0.436^*^	
Body weight	0.076	0.510^**^	
Hand grip strength	0.519^**^	0.478^*^	
DMFT index	− 0.140	0.056	
Maximum occlusal force	0.437^*^	0.211	
Masticatory performance	0.609^**^	0.102	
SNA angle	0.159	− 0.080	
SNB angle	0.412^*^	0.019	
ANB angle	− 0.373	− 0.149	
SN/ML angle	− 0.082	− 0.122	
ML/NL angle	− 0.191	− 0.082	
SN/NL angle	0.132	− 0.065	
U1/NL angle	0.115	0.396^*^	
L1/ML angle	− 0.145	0.419^*^	
Interincisal angle	0.111	− 0.548^**^	
L1 to APg distance	− 0.120	0.504^**^	
Ar to B distance	0.093	0.371	
Overjet	− 0.099	0.176	
Overbite	− 0.070	− 0.671^**^	

^*^ *P* < 0.05; ^**^ *P* < 0.01. DMFT, decayed, missing, and filled teeth

Maximum tongue pressure in the Class I group was significantly positively correlated with hand grip strength, maximum occlusal force, masticatory performance, and the SNB (sella, nasion, B point) angle (all, *p* < 0.05). Maximum tongue pressure in the Class II group was significantly positively correlated with height, body weight, hand grip strength, U1/NL angle, L1/ML angle, and L1 to APg distance (all, *p* < 0.05). By contrast, maximum tongue pressure in the Class II group was significantly negatively correlated with the interincisal angle and degree of overbite (both, *p* < 0.01).

In the Pearson’s correlation analysis, hand grip strength was significantly positively correlated with maximum occlusal force in the Class I group (*r* = 0.503, *p* < 0.01; data not shown), although this was not in the case for the Class II group (*r* = 0.197, *p* = 0.315; data not shown).

Height, body weight, and hand grip strength were significantly positively correlated with each other, with *r* > 0.61 in both the Class I and Class II groups (all, *p* < 0.05; data not shown). In the Class II group, the partial correlation between maximum tongue pressure and hand grip strength after controlling for height and body weight was 0.278, but this correlation was not significant (*p* = 0.17).

## Discussion

In this study, we evaluated tongue pressure-related factors, including maxillofacial morphology and masticatory performance, in cases of Class I and Class II malocclusions during the mixed dentition period. The results showed that the factors associated with tongue pressure were significantly different between the Class I and Class II malocclusion groups.

The present data indicate that skeletal muscle strength (hand grip strength) in the Class II group was significantly lower than that in the Class I group. According to the Japanese Survey on Physical Fitness and Motor Abilities in 2017, the average hand grip strength in 8- and 9-years-old boys was 13.08 and 14.90 kg, respectively, while that in 8- and 9- year-old girls was 12.31 and 14.13 kg, respectively [20]. In this study, the mean hand grip strength in the Class II malocclusion group was lower than the national average for both boys and girls (see Table 1). However, according to the Annual Report of School Health Statistics Research in 2020, the mean height and weight of our participants were within the national average range in both the Class I and Class II groups [21].

Recently, hand grip strength was shown to be positively correlated with maximum occlusal force in adults [22]. Our results for the Class I group are consistent with this. Another study reported that occlusal force was significantly positively correlated with vertical and transverse facial dimensions and significantly negatively correlated with mandibular inclination in young adults [23]. Thus, the skeletal muscle strength may be linked to the power of the masticatory muscles. The morphology of the maxillofacial skeleton to which these muscles are attached may be also directly or indirectly associated with skeletal muscle strength. However, we did not find a significant relationship between the maximum occlusal force and hand grip strength in cases of Class II malocclusion, although the maximum occlusal force in cases of Class II malocclusion was significantly lower than that in cases of Class I malocclusion.

Given the above, we believe that patients with Class II malocclusion may be affected by factors related to low skeletal muscle strength, that do not strongly impact occlusal force.

Consistent with a recent study, we found that the maximum tongue pressure in cases of Class II malocclusion was also significantly lower than that in cases of Class I malocclusion [10]. Bivariate analysis showed that the maximum tongue pressure in the Class II group was significantly positively correlated with body size (height and body weight) and skeletal muscle strength, but after controlling for height and body weight, there was no significant correlation between maximum tongue pressure and hand grip strength. On the other hand, in the Class I group, the maximum tongue pressure was significantly positively correlated with skeletal muscle strength independently of the parameters of body size.

These results suggest that the relationship between maximum tongue pressure and skeletal muscle strength is not direct and may be dependent on body size in cases of Class II malocclusion.

Therefore, we believe that patients with Class II malocclusion with a smaller physique may have a reduced ability to generate high tongue pressure.

In a previous study, the maximum tongue pressure was significantly higher in patients with Class III malocclusion (mean age, 9.1 years) than in those with Class II malocclusion, but there was no significant difference compared to cases with Class I malocclusion. In addition, they reported that a larger SNB angle and smaller ANB angle were related to greater maximum tongue pressure and suggested that the development of tongue pressure may contribute to mandibular growth [10]. Similar findings were seen in our study, but only for cases of Class I malocclusion and not for those with Class II malocclusion.

Generally, both heredity and mouth breathing are epidemiological factors associated with the development of Class II malocclusion in children with mixed dentition [24]. One study reported that tongue pressure was lower in children with mouth-breathing behavior than in children with nasal-breathing behavior [25]. Further, Class II morphology with nasal obstruction and inferior tongue posture was related to narrow maxillary dentition in children [26]. Another study reported a positive correlation between palatal width and maximum tongue pressure in adult patients [27]. These results suggest that mouth breathing may be related to low tongue pressure in patients with Class II malocclusion.

A recent study reported that children with Class III malocclusion (mean age, 9.2 years) had a large tongue volume and small anatomical balance (tongue volume/oral cavity volume), with the reverse true for children with Class II malocclusion [28]. A greater tongue volume to oral cavity volume ratio has been implicated in obstructive sleep apnea, which is accompanied by mouth breathing [29]. Tongue volume and the tongue volume to oral cavity volume ratio may be related to the lingual inclination of the incisors and a deep overbite, which were significantly related to low maximum tongue pressure in cases with Class II malocclusion. In cases of Class II malocclusion, small tongue volume and a large tongue volume to oral cavity volume ratio might also contribute to low maximum tongue pressure. As the ANB angle of Class II malocclusions in this study was > 4° on average, many patients with skeletal maxillary prognathism were included in our study population.

 Therefore, although the causal relationship is not clear, in cases of Class II malocclusion, the maxillofacial morphology and incisor lingual inclination peculiar to maxillary prognathism may hinder the development of tongue pressure, or the underdevelopment of tongue pressure may lead to abnormalities in the inclination of the incisors and other aspects of oral morphology.

A previous study reported that masticatory performance and maximum tongue pressure were significantly positively correlated among children aged 6–12 years [6]. In this study, we found that masticatory performance and tongue pressure were significantly positively correlated in the Class I group but not the Class II group. Additionally, maximum occlusal force and maximum tongue pressure were significantly positively correlated in the Class I group but not the Class II group.

These results suggest that the coordination of maximum tongue pressure and masticatory performance, and the occlusal force, are characteristic findings in Class I malocclusions, and that Class II malocclusions may have other factors related to masticatory performance.

In this study, masticatory performance did not obviously differ between the Class I and Class II groups, unlike maximum occlusal force and maximum tongue pressure, although mean masticatory performance in the Class II group was lower than that in the Class I group. It may be that a larger number of chewing cycles compensated for lower occlusal force and lower tongue pressure in cases of Class II malocclusion.

Therefore, we believe that the masticatory muscle movement in patients with Class II malocclusion may be more active than that with Class I malocclusion.

This study had some limitations. Dentition and maxillofacial features, including soft tissue morphology related to tongue pressure and malocclusion and normal occlusion, could not be clarified. Further studies of factors related to tongue pressure, including the morphology of the tongue and oral cavity in malocclusion and normal occlusion, are needed.

## Conclusions

The results of the present study demonstrated that the factors associated with maximum tongue pressure were clearly different between cases with Class I and Class II malocclusions during the mixed dentition period. Maximum tongue pressure was significantly positively correlated with body size and incisor inclination angle in Class II malocclusions, but with skeletal muscle strength, SNB angle, maximum occlusal force, and masticatory performance in Class I malocclusions.

## Data Availability

The datasets used and analyzed during the current study are available from the corresponding author on reasonable request.

## Abbreviations

SNB: sella, nasion, B point
ICC: intraclass correlation coefficient
DMFT: decayed, missing, and filled teeth
ANB: A point, nasion, B point
